# A high-density consensus map of barley linking DArT markers to SSR, RFLP and STS loci and agricultural traits

**DOI:** 10.1186/1471-2164-7-206

**Published:** 2006-08-12

**Authors:** Peter Wenzl, Haobing Li, Jason Carling, Meixue Zhou, Harsh Raman, Edie Paul, Phillippa Hearnden, Christina Maier, Ling Xia, Vanessa Caig, Jaroslava Ovesná, Mehmet Cakir, David Poulsen, Junping Wang, Rosy Raman, Kevin P Smith, Gary J Muehlbauer, Ken J Chalmers, Andris Kleinhofs, Eric Huttner, Andrzej Kilian

**Affiliations:** 1 Triticarte P/L, PO Box 7141 Yarralumla, Canberra, ACT 2600, Australia; 2 DArT P/L, PO Box 7141 Yarralumla, Canberra, ACT 2600, Australia; 3 School of Agricultural Science, University of Tasmania, PO Box 252-54, Hobart TAS 7001, Australia; 4 Tasmanian Institute of Agricultural Research, PO Box 46, Kings Meadows TAS 7249, Australia; 5 NSW Agricultural Genomics Centre and NSW Department of Primary Industries, Wagga Wagga Agricultural Institute, PMB, Wagga Wagga NSW 2650, Australia; 6 GeneFlow Inc., 14582 Olde Kent Rd., Centreville VA 20120, USA; 7 School of Agriculture, Food and Wine, Plant Genomics Centre, The University of Adelaide, PMB1, Glen Osmond SA 5064, Australia; 8 Dept. Crop and Soil Sciences and School of Molecular Biosciences, Washington State University, Pullman WA 99164-6420, USA; 9 Research Institute of Crop Production, Drnovská 507, 161 06 Prague 6, Czech Republic; 10 Molecular Plant Breeding CRC, WA State Agricultural Biotechnology Centre, Murdoch University, Murdoch, WA 6150, Australia; 11 Department of Primary Industries & Fisheries, Plant Science, MS 508 Warwick, QLD 4370, Australia; 12 Department of Agronomy and Plant Genetics, University of Minnesota, St. Paul, MN 55108, USA

## Abstract

**Background:**

Molecular marker technologies are undergoing a transition from largely serial assays measuring DNA fragment sizes to hybridization-based technologies with high multiplexing levels. Diversity Arrays Technology (DArT) is a hybridization-based technology that is increasingly being adopted by barley researchers. There is a need to integrate the information generated by DArT with previous data produced with gel-based marker technologies. The goal of this study was to build a high-density consensus linkage map from the combined datasets of ten populations, most of which were simultaneously typed with DArT and Simple Sequence Repeat (SSR), Restriction Enzyme Fragment Polymorphism (RFLP) and/or Sequence Tagged Site (STS) markers.

**Results:**

The consensus map, built using a combination of JoinMap 3.0 software and several purpose-built perl scripts, comprised 2,935 loci (2,085 DArT, 850 other loci) and spanned 1,161 cM. It contained a total of 1,629 'bins' (unique loci), with an average inter-bin distance of 0.7 ± 1.0 cM (median = 0.3 cM). More than 98% of the map could be covered with a single DArT assay. The arrangement of loci was very similar to, and almost as optimal as, the arrangement of loci in component maps built for individual populations. The locus order of a synthetic map derived from merging the component maps without considering the segregation data was only slightly inferior. The distribution of loci along chromosomes indicated centromeric suppression of recombination in all chromosomes except 5H. DArT markers appeared to have a moderate tendency toward hypomethylated, gene-rich regions in distal chromosome areas. On the average, 14 ± 9 DArT loci were identified within 5 cM on either side of SSR, RFLP or STS loci previously identified as linked to agricultural traits.

**Conclusion:**

Our barley consensus map provides a framework for transferring genetic information between different marker systems and for deploying DArT markers in molecular breeding schemes. The study also highlights the need for improved software for building consensus maps from high-density segregation data of multiple populations.

## Background

Barley (*Hordeum vulgare *L.) was domesticated approximately 10,000 years ago and stands among the four most important cereal crops today [1]. It has received considerable research attention as a model for genetic analyses. Breeding programs around the world are working towards improved varieties with better quality, disease-resistance and agronomic traits [2,3]. Researchers and breeders have increasingly been adopting molecular markers to identify genomic regions influencing traits and to select for desirable phenotypes based on identified marker-trait associations [4-6]. Several barley consensus maps have been built with gel-based marker technologies such as Restriction Fragment Length Polymorphism (RFLP), Simple Sequence Repeats (SSR) and Amplified Restriction Fragment Length Polymorphism (AFLP) [7-11]. These maps integrate information of markers segregating in different crosses and have provided an important framework for producing and exchanging genetic information among members of the scientific community.

Molecular marker technologies, however, are currently undergoing a transition from largely serial technologies based on separating DNA fragments according to their size (SSR, AFLP), to highly parallel, hybridization-based technologies that can simultaneously assay hundreds to tens of thousands of markers (e.g., Single Nucleotide Polymorphisms or SNPs) [12]. This transition is mostly taking place in biomedicine and plant/animal genomics because SNP assay development is both time and cost-intensive. Notwithstanding the SNP discovery efforts in barley [13,14], practical spin-offs for barley breeding have yet to be generated. Diversity Arrays Technology (DArT) offers a rapid and DNA sequence-independent shortcut to medium-density genome scans of any plant species [15-22]. A single DArT assay simultaneously types hundreds to thousands of SNPs and insertion/deletion polymorphisms spread across the genome. Barley was one of the first species for which DArT markers became available [16]. Since then, approximately 2.3 million data points for 4,000 lines have been generated for barley breeders and researchers at Triticarte P/L.

It is essential to integrate the rapidly growing body of genetic information produced through DArT with the existing genetic knowledge generated through other marker technologies. The key objective of this study, therefore, was to create a "bridge" between DArT and other marker systems in the form of a ~3,000-locus consensus map that co-locates different types of markers. This consensus map was built from the combined set of segregation data of ten different populations assayed with DArT, most of which were also assayed with SSR, RFLP and/or STS markers (Table 1).

**Table 1:** Populations and markers assayed.

**Population**	**Type**	**DH method**	**Size**	**Traits segregating**	**'bPb' DArT markers**	**'bPT' DArT markers**	**Other markers**	**Reference**
Barque-73/CPI71284-48	DH	Anther culture	85	-	530	-	166	Hearnden et al., unpublished
Clipper/Sahara	DH	*Hordeum bulbosum*	88	Cereal cyst nematode resistance, boron tolerance, zinc accumulation, row number, grain color	522	-	293	Karakousis et al. [52]
Dayton/Zhepi2	DH	Anther culture	85	Aluminum tolerance, malting quality	493	-	38	Raman et al., unpublished
Foster/CI4196	F_8–9 _RIL	-	86	*Fusarium *head blight, deoxynivalenol accumulation, spike angle and density, days to heading, plant height, number of rachis nodes	309	-	247	Horsley et al. [50]
Frederickson/Stander^a^	F_4–6 _RIL	-	54	*Fusarium *head blight, deoxynivalenol accumulation, heading date, *vrs1 *locus	380	-	-	Mesfin et al. [48]
Igri/Atlas68^a^	DH	Anther culture	54	*Yd2*, disease tolerance, field performance	480	-	-	Kucera et al., unpublished
Patty/Tallon	F_6 _RIL	-	96	net blotch, leaf rust, kernel discoloration, grain characteristics	257	-	-	Cakir et al., unpublished
Steptoe/Morex	DH	*Hordeum bulbosum*	94	Malting quality, yield, disease resistance, heading date, plant height, lodging, seed weight	483	539	212	Kleinhofs et al. [32]
TX9425/Franklin	DH	Anther culture	89	Waterlogging tolerance, malting quality	370	-	24	Li et al. [49,51]
Yerong/Franklin	DH	Anther culture	180	Disease resistance, waterlogging tolerance	450	-	22	Li et al., unpublished

^a ^Part of the DNA samples stored in 96-well microtiter plates got cross-contaminated during shipment as a result of insufficient sealing of the plates, and the corresponding DArT assays, with an excess of '1' scores, had to be discarded.

In the course of constructing this consensus map it became clear that the performance of available software for building consensus maps was insufficient for our high-density dataset. As a consequence, this study has a second, methodological component, in which we identify some insufficiencies of existing mapping software and explore the performance of alternative map construction strategies in order to highlight the need for software improvements in this area.

## Results and discussion

### Software performance with high-density linkage data

JoinMap 3.0 is one of the most commonly used programs for constructing linkage maps for plant populations. Importantly, it appears to be the only software option for building a consensus map from the integrated dataset of multiple populations derived from independent crosses between different pairs of parents [23,24]. We found, however, that this program reproducibly generated erroneous results with our high-density datasets. Problems with using JoinMap to analyze high-density datasets have apparently been encountered by others as well [25,26]. Inspection of graphical genotypes for the locus order generated by the program revealed considerable numbers of misplaced loci and inversions of blocks of loci, which introduced artificial crossovers and inflated maps.

As an example, we built individual maps for the high-density DArT datasets of three populations and evaluated map quality by computing the sum of adjacent recombination fractions (SARF), a sensitive quantifier of map expansion caused by a suboptimal locus order [27]. Compared to a similar set of maps constructed with a well-performing locus-ordering algorithm (RECORD) [26], the linkage groups built with JoinMap at the preset default settings of the program were inflated by 70 ± 76%. The program furthermore failed to incorporate 15 ± 14% of loci into linkage groups, although some of these loci co-segregated with other loci that were incorporated (Table 2).

**Table 2:** Performance of JoinMap 3.0 with high-density linkage data.^a^.

**Dataset**	**LOD threshold**	**Unincorporated markers**	**Map inflation**^b^	**Split linkage groups**
Complete	1.0	15 ± 14% (range: 0–54%)	70 ± 76% (range: 1–256%)	0%
Complete	2.0	14 ± 12% (range: 1.2–36%)	61 ± 82% (range: 5–279%)	38%
Binned	1.0	0.8 ± 1.8% (range: 0–7%)	43 ± 71% (range: 1–269%)	0%
Binned	2.0	0.2 ± 0.2% (range: 0–0.2%)	2.8 ± 2.3% (range: 0–7.5%)	38%

^a ^All values reported are means ± SD across 21 linkage groups (7 chromosomes × 3 populations) or a subset of them in case there was insufficient linkage information for map construction. The populations used for this test were Clipper/Sahara, Dayton/Zhepi2 and Steptoe/Morex. Only DArT markers were used, in order to disentangle the potential effect of (hypothetical) DNA sample tracking errors from software performance. The LOD threshold was varied, while the other program settings were held constant (REC = 40; ripple after each locus; threshold for marker removal = 5). Decreasing the REC threshold had a similar effect as increasing the LOD threshold (data not presented).

^b ^Map inflation values are the percent increase in SARF for the locus order reported by JoinMap compared to the order reported by RECORD. If JoinMap did not incorporate all loci into a map, only the set of incorporated loci were analyzed with RECORD. For unknown reasons, heavily inflated maps with a large number of misplaced loci were often reported by JoinMap to be shorter than maps constructed from the same datasets using JoinMap settings that produced a close-to-optimum locus order.

Increasing the stringency of the program's settings marginally improved the results. More stringent thresholds, however, also made it necessary to split linkage groups into subgroups if the remaining linkage information was insufficient to construct a map (Table 2). In any case, the relationship between the program's settings and the degree of map expansion appeared to vary across different linkage groups, thus requiring a separate optimization of the settings for each linkage group (data not presented). Given the large datasets of this study such a case-by-case optimization would not have been feasible because the computation time of the program is proportional to the forth power of the number of markers [26].

We tested an alternative way of improving the performance of JoinMap: collapsing co-segregating markers into 'bins' (unique loci) with a purpose-built perl script and only using a single representative marker per bin for map construction. This approach almost eliminated the problem of non-incorporated markers and reduced the degree of map expansion to some extent (Table 2). A combination of binning and more stringent program settings reduced the degree of map expansion to a negligible level (2.8 ± 2.3%), but had the side effect of breaking up linkage groups (Table 2). Among the 21 linkage-group comparisons, we did not find any case where JoinMap produced a shorter map than RECORD.

### Component maps of individual populations

**Figure 1:**
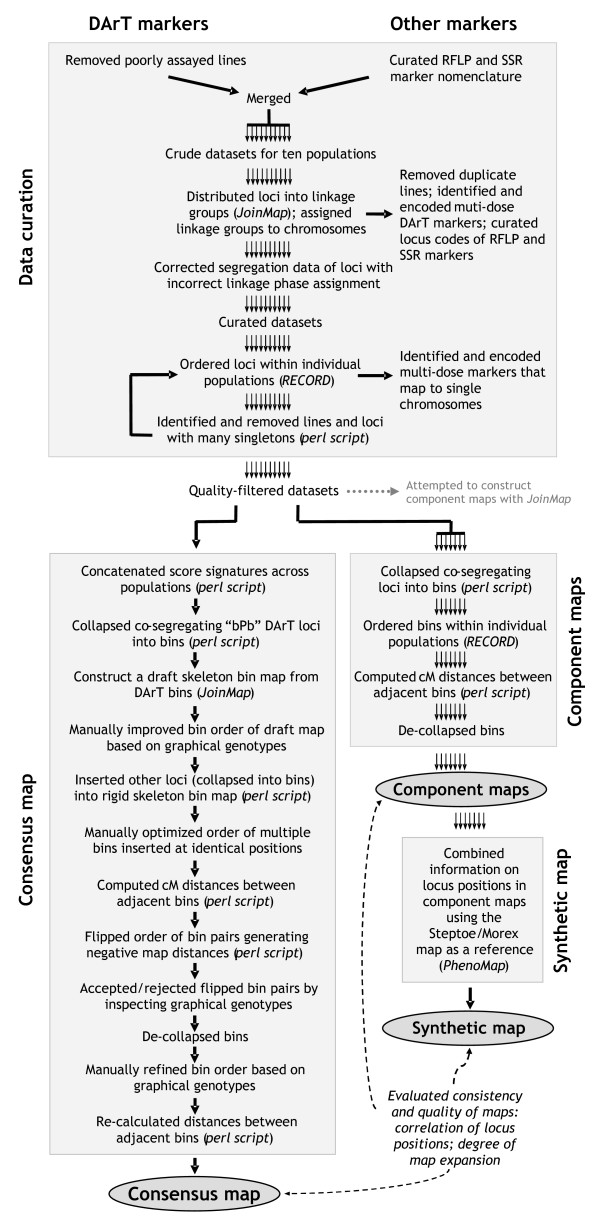
**Schematic outline of map-building strategies used in this study**. Pilot maps were built for each of the ten populations separately to flip the phase of loci assigned to the wrong phase, to identify multi-locus markers, and to remove loci and lines with excessive numbers of singletons (apparent double crossovers). The quality-filtered datasets were then used to build seven 'component' maps for individual populations with sufficient numbers of lines and loci. The integrated dataset of all ten populations was used to build a consensus map. The quality of the locus order of the consensus map was evaluated by comparison against the order of loci in the component maps and a 'synthetic map' derived from the component maps.

Having established the superior performance of RECORD, we used a combination of RECORD and a purpose-built perl script to construct pilot maps of individual populations. The graphical genotypes of these maps were then investigated to identify 'singletons' (apparent double crossovers) pointing to potential genotyping errors [28,29]. We did not replace individual singletons with missing calls because of the possibility of introducing a bias towards a particular (but not necessarily correct) locus order and because recombination events in recombinant inbred lines (RIL) (Foster/CI4196, Frederickson/Stander and Patty/Tallon populations) could be close to each other as a result of multiple rounds of meioses. Instead we completely removed a limited number of less reliable markers (DArT: 4.0%; non-DArT: 6.8%) and lines (0.4%) that had an excessive number of singletons (Figure 1).

**Figure 2:**
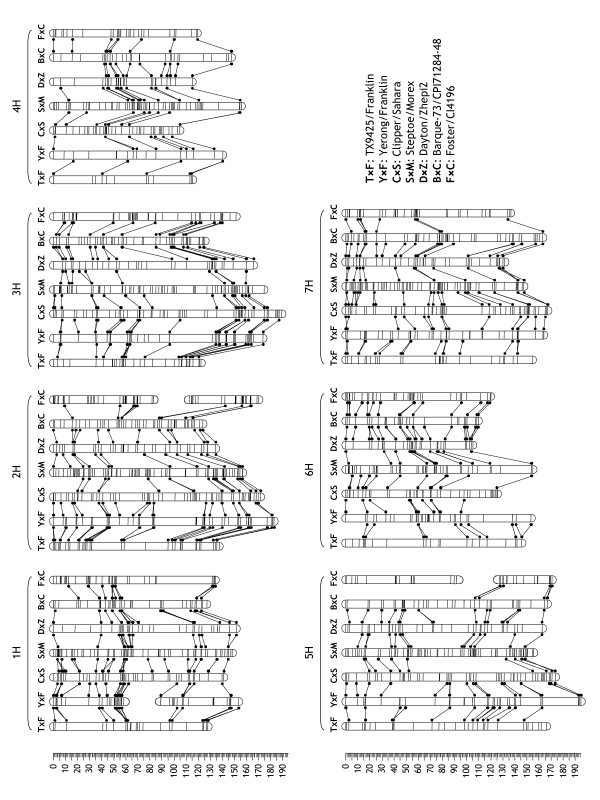
**Colinearity of locus order in component maps**. Loci in component maps are displayed schematically by horizontal lines across the bars representing chromosomes. Loci that are common between adjacent pairs of populations are depicted by dots and connected by lines [30].

We then re-constructed component maps from seven quality-filtered datasets that had sufficient numbers of lines and markers to build a reliable linkage map. The datasets contained between 394 (TX9425/Franklin) and 1,232 loci (Steptoe/Morex) and between 85 and 180 lines (Tables 1 and 3). The lengths of the resulting maps varied between 964 and 1,073 cM (1,030 ± 60 cM; mean ± SD; Table 3). The order of loci that were common among the maps was very similar (Figure 2; Additional Files 1, 2). The method of doubled haploid (DH) production appeared to have a significant effect on the length of the resulting component maps (*p *< 0.016 for a two-tailed *t *test; Table 3). Populations produced with the *Hordeum bulbosum *method, which are derived from meioses that lead to female gametes, tended to produce longer maps than anther culture-derived populations, the products of male meioses. This trend does not coincide with a previous study in which anther-derived populations showed higher recombination rates in distal chromosome areas [31]. It is possible that suppression of recombination in wide crosses was a more important influencing factor because map lengths were negatively correlated (*r *= -0.51) with the number of 'bPb' DArT markers scored in different populations, an approximate measure of the genetic distance between parents.

**Table 3:** Statistics of selected component maps.^a^.

	**B**/**C**	**C**/**S**	**D**/**Z**	**F**/**C**	**S**/**M**	**T**/**F**	**Y**/**F**	**Average ± SD**
**Number of loci**	696	814	531	552	1,232	394	472	670 ± 285
**Number of bins**^b^	289	357	242	309	508	185	262	307 ± 104
**Inter-bin distance (cM)**								
**Average**	3.4 ± 3.8	3.1 ± 3.4	4.1 ± 4.2	2.7 ± 4.7	2.2 ± 2.7	5.4 ± 6.6	4.2 ± 5.6	3.6 ± 1.1
**Median**	2.1	2.0	2.7	1.0	1.2	3.2	1.5	2.0 ± 0.8
**Map length (cM)**	964	1,073	967	1,066	1,093	970	1,072	1,031 ± 60

^a^Abbreviations of maps: B/C, Barque-73/CPI71284-48; C/S, Clipper/Sahara; D/Z, Dayton/Zhepi2; F/C, Foster/CI4196; S/M, Steptoe/Morex; T/F, TX9425/Franklin; Y/F, Yerong/Franklin.

^b ^Co-segregating loci were collapsed into bins (unique loci) at the population-level, i.e. without concatenating segregation signatures across populations.

### A consensus map from the combined datasets

**Figure 3:**
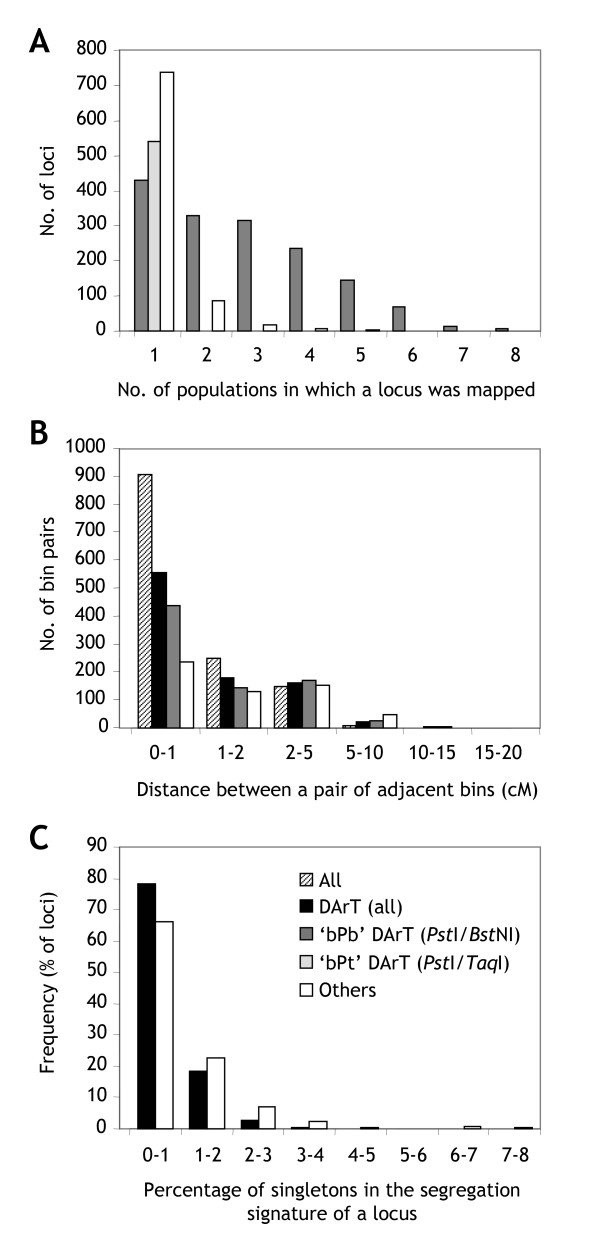
**Consensus map features by marker type**. **(A) **Frequency with which individual markers were mapped in the ten populations. The 'bPb' DArT markers from the *Pst*I*/Bst*NI representation (assayed across all populations) and the 'bPt' markers from the *Ps*tI(*Taq*I) representation (only assayed in the Steptoe/Morex population) were separately compared against other markers (SSR, RFLP, STS). **(B) **Map resolution. Loci from each of four datasets (all markers, all DArT markers, 'bPb' DArT markers, other markers) were collapsed into bins by comparing their segregation signatures across populations**. **The bins were arranged according to the consensus map order, and the distances between pairs of adjacent bins were calculated. **(C) **Map quality. Loci from two datasets (all DArT markers, other markers) were jointly collapsed into bins by comparing their segregation signatures across populations**. **The bins were arranged in the order of the consensus map, and the number of singletons for the locus with the highest call rate within each bin was counted and expressed as a percentage of the number of genotype calls.

We built an initial draft of a consensus map with JoinMap using a limited set of quality-filtered markers under conditions that were likely to minimize the number of misplaced loci (Table 2). We selected the set of 1,546 'bPb' DArT markers for this purpose. This set of markers was assayed across all populations and contained many good-quality anchors bridging populations. Almost three quarters of the markers (1,117 of 1,546) segregated in two or more crosses and more than half of them (795) in three or more (Figure 3A). Within the populations in which they were polymorphic, the vast majority of the 'bPb' markers (94%) were scored with a call rate of >90%. Only lower-quality markers, which tended to have a smaller difference in hybridization intensity between the two allelic states, had lower call rates (Additional File 3).

Building an initial 'skeleton' map from the 'bPb' DArT markers also minimized the chance of human error impacting on map quality for the following reasons: (1) a single aliquot of each genomic DNA sample was simultaneously assayed for the whole set of 'bPb' DArT markers, which eliminated the possibility of (hypothetical) DNA sample tracking errors impacting the integrity of data assembled from separate marker assays; and (2) the capture of segregation data for DArT markers from microarray images was fully automated, thus eliminating the risk of human errors when linking segregation data to marker names.

To minimize software-induced map inflation (Table 2), we collapsed the 1,546 'bPb' markers into 959 bins based on their segregation signatures concatenated across populations and used moderately stringent program settings to assemble a DArT skeleton bin map with JoinMap (LOD = 2; REC = 35). The program nevertheless failed to incorporate between 10 and 20% of the loci of each chromosome, although virtually all of them were later confirmed to perfectly fit into the consensus map. The program also misplaced a significant number of loci. We therefore improved the locus order manually by inspecting graphical genotypes (see *Methods *section entitled "DArT skeleton bin map"). SSR, RFLP and STS markers, as well as a second set of 'bPt' DArT markers from a different genomic representation, were predominantly assayed in one or two populations only (Figure 3A; Additional File 4). These markers were incorporated into the DArT consensus framework using a purpose-built perl script. Subsequently, we computed map distances and refined the locus order with other purpose-built perl scripts (Figure 1). Additional File 5 contains the segregation data of all loci arranged in the order of the final consensus map.

### Consensus map features

**Figure 4:**
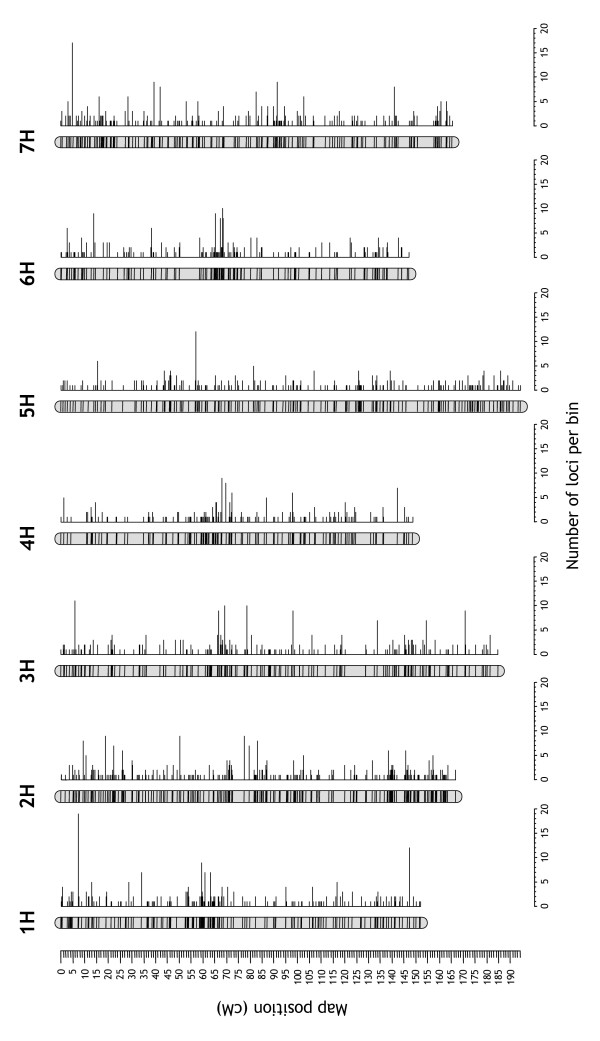
**Schematic view of the consensus map**. The 2,935 loci of the consensus map were collapsed into 1,629 bins by comparing their segregation signatures across populations. Each bin is represented by a horizontal line across a chromosome. The lengths of the horizontal lines to the right of each chromosome depict the number of co-segregating markers within each bin.

The consensus map comprised 2,825 markers mapped to a total of 2,935 loci (2,085 DArT and 850 other loci) (Figure 4; Additional Files 4, 6). This number is considerably larger than the number of markers in previously published consensus maps (587–1,536) [7-10,13]. Only a simultaneously developed SSR consensus map will contain a similar number of markers [11; Andreas Graner, personal communication]. Because of the high multiplexing level of DArT, the production of the more than half a million data points underlying our consensus map has taken only a fraction of the effort that would be required to generate a comparable SSR dataset. Currently, a single operator can produce such a dataset within two weeks; an improved assay format under development is going to reduce this time to four days or less.

On the average, each chromosome contained 298 DArT and 121 non-DArT loci. The number of DArT loci per chromosome ranged from 148 (4H) to 373 (7H). The number of non-DArT loci ranged from 92 (6H) to 160 (2H) (Additional File 7). The number of DArT loci per chromosome probably reflects the distribution of DNA polymorphism across chromosomes more accurately than the numbers of non-DArT loci, because researchers may have targeted particular genomic regions of interest using selected SSR or RFLP markers.

#### Coverage

The consensus map spanned a total length of 1,161 cM. Chromosome sizes ranged from 147.1 cM (6H) to 194.2 cM (5H) (Figure 4). The 'bPb' DArT markers alone spanned 98.1% of the total length of the consensus map. Addition of a second set of DArT markers ('bPt' markers) increased coverage to 99.7%. The combination of all non-DArT markers resulted in a coverage of 96.9% (Additional File 7). A single 'bPb' DArT assay, therefore, provides slightly greater genome coverage than the set of 850 SSR and RFLP markers included in this study.

The map had no gap larger than 10 cM and only nine gaps between 5 and 10 cM (3H, 4H, 6H and 7H). The DArT subset of markers generated a consensus map with a single gap between 15 and 20 cM (4HS) and five gaps between 10 and 15 cM on chromosomes 3H, 4H and 5H (the set of 'bPb' markers alone had an additional gap of this size on 4H). Chromosome 4H has previously been noted by others to be less polymorphic than the others [8,9,32]. Non-DArT markers on their own resulted in a map with one gap between 15 and 20 cM (3HL) and two gaps between 10 and 15 cM on chromosome 6H (Figure 3B). The smaller number of 10–15 cM gaps in the non-DArT dataset may reflect targeted efforts to fill gaps in component maps with selected SSR or RFLP markers.

#### Resolution

The average resolution of the consensus map was evaluated by collapsing co-segregating loci into bins and calculating the average distances between adjacent bins. The 2,935 loci of the whole dataset could be distributed into 1,629 bins with an average inter-bin distance of 0.71 ± 1.01 cM (median = 0.32 cM). This resolution was only moderately greater than the resolution obtained with DArT loci alone (1.03 ± 1.59 cM; median = 0.40 cM). The set of 'bPb' DArT markers, which were simultaneously assayed in a single reaction, provided a resolution of 1.20 ± 1.83 cM (median = 0.44 cM). Non-DArT markers on their own produced a map with a resolution of 1.91 ± 2.07 cM (median = 1.26 cM; Additional File 7).

#### DArT marker redundancy

The DArT markers were originally obtained by cloning random fragments of genomic representations [16], a process that introduces some degree of marker redundancy. The 1,546 'bPb' DArT loci could be collapsed into 959 bins, suggesting a redundancy level of 38%. Co-segregating 'bPb' DArT markers, however, were not necessarily multiple copies of a single marker because more than 100 of the 'bPb' DArT bins contained markers that were in the opposite allelic phase in some crosses (data not presented). On the other hand, a small number of genotyping errors may have prevented multiple copies of single markers from being collapsed into bins. Therefore, it may not be surprising that the redundancy estimate obtained from marker segregation analysis was quite similar to the preliminary estimate obtained by clustering the DNA sequences of DArT markers (data not presented).

Marker redundancy is a transient feature of DArT array development, which proceeds by consolidating the most informative clones in new arrays of increasing information content [21]. During this process redundant markers are excluded from the final genotyping array.

#### Marker-dense regions

**Figure 5:**
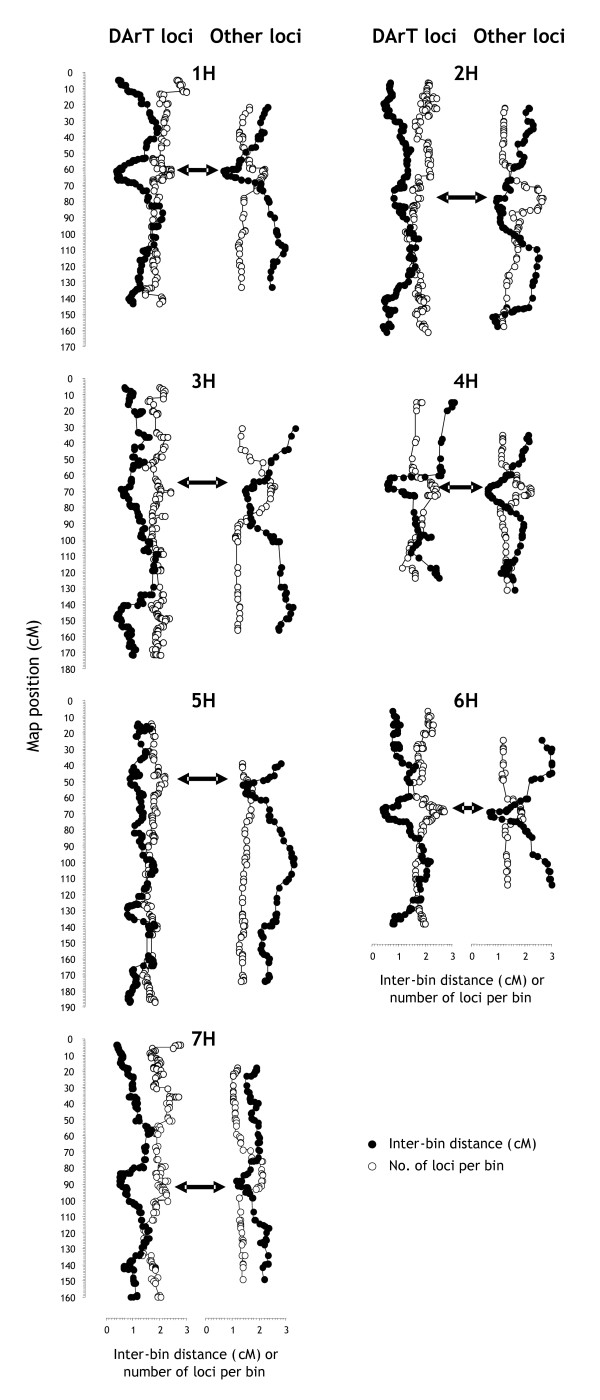
**Visualization of marker-dense regions in barleychromosomes**. DArT and non-DArT loci were separately collapsed into bins by comparing their segregation signatures across populations. The bins of each dataset were arranged in the order of the consensus map. The number of loci per bin and the distance between adjacent bins (inter-bin distance) were then averaged across a 19-bin sliding window that was moved across each chromosome. Approximate centromere positions are indicated by horizontal two-sided arrows.

Markers sometimes tend to cluster, either as a consequence of an uneven distribution of recombination events along chromosomes [33] or because they preferentially survey DNA polymorphism that is unevenly distributed along chromosomes [34,35]. Regions of the consensus map with high marker densities were visualized by plotting local averages of inter-bin distances and the number of loci per bin along chromosomes (Figure 5). Both DArT and non-DArT loci showed a moderate tendency to cluster around centromeres as can be deduced from the shorter inter-bin distances and the larger numbers of loci per bin in the vicinity of centromeres. This clustering tendency, however, was nowhere near as pronounced as, for example, for AFLP markers based on methylation-insensitive restriction enzymes [36]. Given the different polymorphism-detection principles of DArT, SSR and RFLP markers, we suggest that the centromeric clustering largely reflects centromeric suppression of recombination [33,37]. Centromeric clustering, however, was less pronounced in chromosome 5H, a feature that was previously noted by others [8].

In some chromosomes the density of DArT markers also appeared to be higher in distal regions (1H, 2H, 6H and 7H). This pattern may reflect a moderate bias of *Pst*I-based DArT markers towards gene-rich, hypomethylated areas in telomeric chromosome regions [38], a pattern we also observed in wheat [21]. A preliminary analysis of the DNA sequences of the 'bPb' DArT markers indeed suggests that the majority of them are derived from the genespace (data not presented).

#### Multi-locus markers

Markers mapping to more than one locus, if not recognized, can be a confounding factor in the process of building a consensus map. Among the 1,523 mapped 'bPb' DArT markers, only 21 (1.4%) mapped to two different loci in different populations and one (0.2%) mapped to three different loci (Additional File 8). The loci of multi-locus DArT markers were usually located on different chromosomes; two of them, however, mapped to loci within a single chromosome (1H and 4H; Additional File 4). Multi-locus markers were more common among other marker types. The set of 753 non-DArT markers contained 66 markers (8.8%) that mapped to 2 to 8 different loci each (Additional File 4, 8). The difference in frequency of multi-locus markers between DArT and other marker types reflects the fact that as a hybridization-based method DArT inherently selects against multi-locus markers. The hybridization intensities measured for such markers are a difficult-to-resolve mixture of the contributions of several loci, which makes them appear as 'monomorphic'.

#### Residual singletons

A good indicator of the quality of a linkage map constructed from DH populations is the frequency of singletons (apparent double crossovers), which are often due to genotyping errors [28,29]. The frequency of singletons was calculated from the 1,629-bin dataset containing all types of markers by using a purpose-built perl script. Approximately 0.35% of all calls for non-DArT loci were singletons. DArT loci generated singletons at a rate of approximately 0.20% (Additional File 5). The majority of the loci with singletons introduced less than one singleton per one hundred calls (Figure 3C). Not surprisingly, high-quality DArT markers, which tend to have allelic states with more contrasting hybridization intensities, generated fewer singletons: the correlation between the across-population average of the marker quality parameter and the percentage of singletons in the concatenated segregation signature was -0.40. The Frederickson/Stander population, and to a lesser extent the Igri/Atlas68 population, contained larger numbers of singletons, presumably because some of the DNA samples got cross-contaminated during shipment as a result of insufficient sealing of microtiter plates (data not presented; see *Methods *and Table 1).

Singletons presumably were not only the result of genotyping errors. The comparatively large distances between adjacent loci on chromosome 4H, true double crossovers events in the RIL populations (Foster/CI4196, Frederickson/Stander and Patty/Tallon), unstable methylation patterns [16] and possibly gene conversion events [39] may have introduced some singletons. The reported singleton rates, therefore, almost certainly overestimate the error rates of marker assays. The overestimation of genotyping error rates, however, was to a degree offset by having removed low-quality markers during the construction of pilot maps (see *Methods *section). The frequency of DArT singletons, therefore, is in good agreement with the previous 0.2% estimate of the error rate of DArT assays [16,21].

### Comparison with component maps

An alternative way to evaluate the quality of a consensus map is to compare the locus arrangement of the consensus map (optimized at the multi-population level) with the arrangement of loci in the component maps (each one optimized separately). We selected seven populations with sufficient numbers of lines and loci for this comparison (Table 3).

To quantify the consistency of locus order between the two different types of maps, unique loci of each of the seven datasets were alternatively arranged according to the consensus or the component map to compute two alternative sets of locus positions per dataset. The correlation coefficients for the alternative sets of locus positions ranged from 0.9998 ± 0.0003 (1H) to 0.99996 ± 0.00006 (3H) (means ± SD across seven populations). We conclude that the order of loci in the consensus map properly reflects the arrangement of loci in the individual component maps.

As a separate indicator of the quality of the consensus locus order, we also quantified the degree to which component maps expanded if their loci were arranged according to their order in the consensus map. Chromosome lengths computed with the algorithm of Lalouel [40] (also used in JoinMap 3.0) hardly showed any expansion: 0.34 ± 0.43% (mean ± SD across populations). The sum of adjacent recombination fractions (SARF), a more sensitive indicator of map expansion caused by suboptimal marker positioning, revealed a minor degree of expansion of 5.2 ± 2.9% (mean ± SD across populations). This is not surprising because some residual genotyping errors can cause an incorrect locus order to appear superior to the correct order, which can happen more easily if only the segregation data of a single population are taken into account.

Both the indicator of locus order consistency and the degree of map expansion were closely associated with the fraction of DArT loci in the component datasets. Datasets dominated by DArT markers showed more favourable values (Additional File 9). These trends probably reflect two factors. First, non-DArT markers were, on the average, assayed in fewer populations than DArT markers (Figure 3A). Their positions on the consensus map, therefore, were more ambiguous, particularly if they were located in regions where component maps differed in length. Second, the initial draft of the consensus map was built from the 'bPb' DArT markers only. Any (hypothetical) error in DNA sample tracking between DArT and non-DArT marker assays would have introduced artificial crossovers which may have differentially impacted on the accuracy of locus ordering in component maps and the consensus map (see previous paragraph).

### Comparison with a synthetic map constructed from component maps

**Figure 6:**
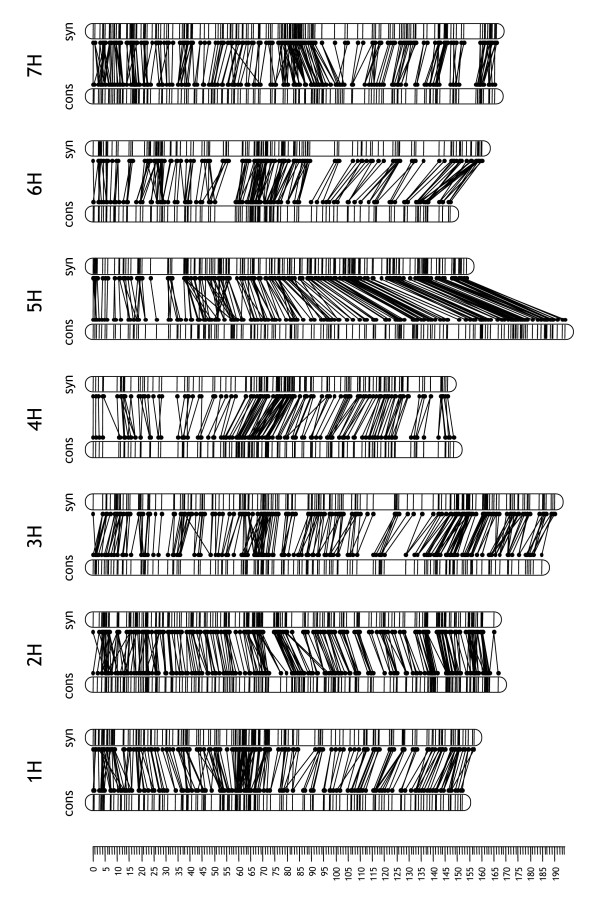
**Alignment of the consensus map with a syntheticmap**. Comparison of locus positions between the JoinMap/perl consensus map ('cons') and a synthetic map built with PhenoMap software using the Steptoe/Morex map as a reference map ('syn'). The position of each locus in the two maps is highlighted by a pair of dots connected by a line [30].

Consensus maps typically are constructed using one of the following two alternative strategies. In strategy I (used in this study) the segregation data from several populations are simultaneously considered to compute the optimum order of loci. In strategy II, various subsets of loci typed for different populations are separately ordered to construct component maps. Subsequently, a 'synthetic' map is constructed by merging information on locus positions from component maps. We investigated the relative efficacy of the two approaches by quantifying how similar a synthetic map was to the consensus map built using a combination of JoinMap and purpose-built perl scripts. We built three alternative synthetic maps with PhenoMap software (GeneFlow Inc., Centreville, VA) to determine the impact of the program settings on the results. Pairwise correlation coefficients for locus positions of the three alternative maps varied between 0.980 (4H) and 0.999 (1H). The synthetic map obtained using the map with the largest number of loci (Steptoe/Morex) as a 'base' (reference) map, was most similar to the JoinMap/perl consensus map, although there were notable differences in chromosome lengths (Table 4; Figure 6).

**Table 4:** Comparison between the JoinMap/perl consensus map and three alternative synthetic maps built with PhenoMap.

	**Base map**		
	
	**Steptoe/Morex**	**Yerong/Franklin**	**Selected by PhenoMap**^a^
**Correlation between locus positions in synthetic maps and locus positions in the consensus map**^b^			

Mean ± SD	0.996 ± 0.004	0.992 ± 0.014	0.995 ± 0.006
Range	0.988–0.998	0.959–0.998	0.982–0.998

**Chromosome lengths in synthetic maps as a percentage of the lengths in the consensus map**^b^			

Mean ± SD	98.7 ± 9.4%	103.9 ± 8.4%	96.5 ± 6.2%
Range	79.1–108.8%	95.2–119.2%	86.0–103.2%

^a ^The base (reference) maps selected by PhenoMap were: Clipper/Sahara (1H), Yerong/Franklin (2H), Clipper/Sahara (3H), Dayton/Zhepi2 (4H), Barque-73/CPI71284-48 (5H, 6H) and Dayton/Zhepi2 (7H).

^b ^The values reported are averages across chromosomes. Eighty-five loci not assayed in the seven populations used to construct the synthetic maps (Table 3) were removed from the JoinMap/perl consensus map before the comparison.

We selected the Steptoe/Morex-based synthetic map for a quality comparison with the JoinMap/perl consensus map. As for the JoinMap/perl consensus map, we quantified the similarity of locus positions between synthetic map and component maps. The resulting correlation coefficients ranged from 0.9994 ± 0.0012 (6H) to 0.99992 ± 0.00007 (5H) (means ± SD across seven populations), a marginally lower range of values compared to the JoinMap/perl consensus map (see previous section). The locus order of the synthetic map was only slightly less optimal than the locus order of the consensus map: the SARF index indicated a map inflation of 7.0 ± 1.6% compared to 5.2 ± 2.9% for the consensus map (means ± SD across seven populations; see previous section). We conclude that the synthetic map built with PhenoMap is reasonably consistent with the consensus map in terms of locus order (Figure 6). The distances between loci, however, appeared to be somewhat less accurate (see the comparatively low correlation coefficients for locus positions in Table 4). The marginally lower quality of the locus order and the less precise map distances, however, are more than offset by the ease and speed of map construction (approximately 10 min of computation time) compared to the alternative JoinMap/perl method (several months of semi-manual data processing).

### Associations between DArT markers and agricultural traits

**Figure 7:**
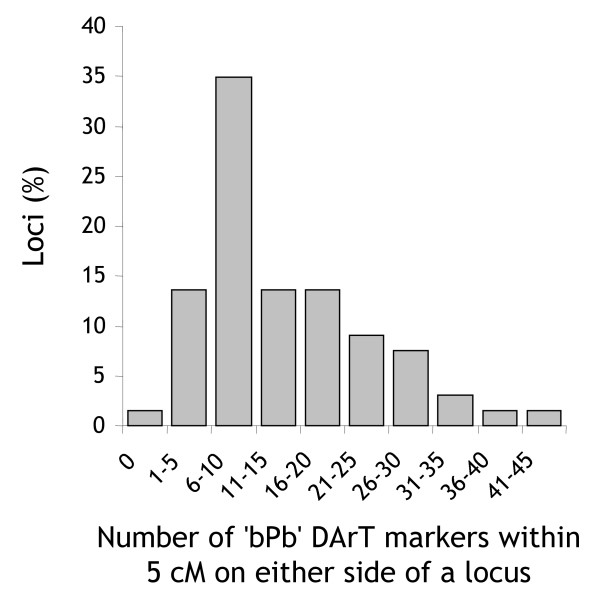
**Number of 'bPb' DArT loci linked to loci affecting agricultural traits**. Histogram of the number of 'bPb' DArT loci within 5 cM on either side of 66 loci affecting agricultural traits. The positions of these loci were defined by SSR, RFLP or STS markers that were incorporated into the consensus map and had previously been identified to be closely linked to agricultural traits (Additional Files 11, 12).

We sampled non-DArT markers from the consensus map that previously had been reported as linked to traits of agricultural relevance (66 loci in total). On the average there were 14 ± 9 DArT markers within 5 cM on either side of these loci tagged by non-DArT markers (range: 0–41 DArT markers). Approximately 95% (63/66) of the loci had at least three and more than half of the loci (56%) had more than ten DArT markers in their vicinity (Figure 7). Only the *β*-amylase locus on 4HS tagged by SSR marker HVM40 was more than 5 cM (7.3 cM) from the closest DArT marker. The average number of DArT markers around trait-influencing loci varied among chromosomes from 8 (4H) to 41 (6H) (Additional File 10).

Additional Files 11 and 12contain a tabular and a graphical summary of DArT markers in the vicinity of loci affecting agricultural traits. The genetic knowledge encapsulated in this table provides a framework for validating and implementing DArT genome profiles to assemble a combination of favorable alleles into improved lines. The collocation of DArT and SSR markers on the consensus map also enables barley breeders to quickly identify target loci through whole-genome DArT scans and then use SSR markers from the same regions for marker-assisted selection.

### Utility of the consensus map for breeding and genomics applications

The consensus map provides a reference for rapidly profiling the genetic diversity within and among the genomes of cultivars, parental lines and new germplasm. It facilitates identification of co-ancestral or genetically distinct genomic regions and enables the detection of genome rearrangements such as translocations (Ignacio Romagosa, personal communication). The positional information attached to DArT markers is going to aid the introgression of novel alleles from wild relatives and to increase the precision with which introgressed fragments can be manipulated (selection for specific recombination events or alien fragment sizes) [41]. Whole-genome selection in the context of improving complex traits and ideotype-breeding strategies is also going to benefit from the consensus map [42,43]. The marker density of the consensus map would allow tighter marker-trait associations than the resolution levels achieved in typical QTL studies. Depending on population history, association mapping approaches could potentially have a higher resolution [44,45] and are going to benefit from a consensus map with many precisely ordered loci.

Different marker applications, however, require different marker densities. The DNA sequences of DArT clones could be used to convert DArT markers to single-marker assay formats for applications in breeding programs. The number of loci targeted by marker-assisted selection, however, is on the rise in many barley breeding programs. A single assay covering a 'standard' set of agriculturally important loci may soon be more cost-effective than 'mixing and matching' single-marker assays. We are therefore developing a medium-plex assay format to cost-effectively deploy DArT markers from approximately 30 key loci in marker-assisted foreground selection programs. Higher densities, on the other hand, could be achieved for chromosome-landing [46] and map-based cloning approaches by simply pyramiding DArT markers from several genomic representations. In this context, we are also working towards integrating DArT markers with other high-throughput marker technologies such as SNP [14].

## Conclusion

The consensus map built in this study co-locates DArT markers with previously mapped SSR, RFLP and STS markers and loci influencing agricultural traits. It provides a framework for deploying DArT markers in molecular breeding schemes, for transferring genetic information between different marker systems and for integrating DArT markers with other genomic resources.

The study has also highlighted an increasing mismatch between our ability to rapidly genotype a large number of mapping populations and the performance of available software tools to construct a consensus map. While from a statistical point of view it is preferable to build a consensus map *de novo *from the integrated set of segregation data, it currently appears preferable to build a synthetic map from separately constructed component maps instead; at least until improved or alternative software options become available [24,48].

## Methods

### Barley crosses

This study was based on segregation data from seven populations DH lines and three populations of RIL. With the exception of Barque-73/CPI71284-48, Dayton/Zhepi2, Igri/Atlas68, Patty/Tallon and Yerong/Franklin, the populations had been developed in the context of previously reported studies (Table 1) [32,48-52].

### Marker assays

All ten populations were genotyped with an identical set of DArT markers from a *Pst*I*/Bst*NI representation ('bPb' markers) in a total of 1,050 DArT assays (20 parental and 1,030 progeny assays). The Steptoe/Morex population was also assayed with a second set of DArT markers from a *Pst*I*/Taq*I representation ('bPt' markers). The DArT data for the Steptoe/Morex and the TX9425/Franklin populations have been reported elsewhere [16,51]. The corresponding microarray images, however, were re-analyzed for this study using an improved version of DArTsoft (DArT P/L, Canberra, Australia) with a slightly relaxed marker quality threshold to score a larger number of markers. The other eight populations were genotyped with DArT for this study.

Seven of the ten populations were also genotyped with other types of markers such as SSR, RFLP and/or STS markers, partly in the context of other ongoing studies (Hearnden et al., unpublished data) [32,48,50,52].

#### DArT

DArT assays were essentially performed as described previously [16,21]. Briefly, 20–100 ng of genomic DNA was digested with two units of *Pst*I and two units of *Bst*NI (NEB, Beverly, MA). A *Pst*I adapter (5'-CAC GAT GGA TCC AGT GCA-3' annealed with 5'-CTG GAT CCA TCG TGC A-3') was ligated to the digested DNA with T4 DNA ligase (NEB). A 1-μL aliquot of the ligation product was used as a template in a 50-μL amplification reaction with DArT-*Pst*I primer (5'-GAT GGA TCC AGT GCA G-3') under the cycling conditions described by Wenzl et al. [16]. The resulting genomic representations were concentrated tenfold by isopropanol precipitation and denatured at 95°C for 2 min. The representations were then labelled with 0.1 μL of Cy3-labelled dUTP (Amersham Biosciences, Castle Hill, NSW, Australia) using the exo^- ^Klenow fragment of *Escherichia coli *DNA polymerase I (NEB) and random decamers for priming. Labelled representations were added to 50 μL of a 50:5:1 mixture of ExpressHyb buffer (Clontech, Mountain View, CA, USA), 10 g L^-1 ^herring sperm DNA (Promega, Annandale, NSW, Australia), and the carboxy-fluorecein (FAM)-labelled polylinker fragment of the plasmid that was used for library preparation [21]. (The hybridization signal for the polylinker fragment was subsequently used by DArTsoft to determine for each clone the amount of DNA spotted on the array; see next paragraph).

The hybridisation mixtures were denatured and hybridized to DArT microarrays which contained 2,304 polymorphism-enriched clones from a *Pst*I*/Bst*NI genomic representation prepared from a mixture of barley cultivars [16]. After overnight hybridization at 65°C, the microarray slides were washed according to Jaccoud et al. [15] and scanned on a Tecan LS300 confocal laser scanner (Grödig, Salzburg, Austria). The microarray images were analyzed with DArTsoft (version 7; DArT P/L, Canberra, Australia), a purpose-built software package. The program measured the relative hybridization intensities (Cy3/FAM) of each clone across all slides, identified clones with variable hybridization intensity (i.e., DArT markers), and used a fuzzy-clustering algorithm to score the corresponding DNA fragments in the genomic representations as present or absent (Cayla et al., in preparation). Individual genotype calls were classified as missing if none of the probabilities of belonging to a particular allelic state (present vs. absent) surpassed 0.8. The quality of each marker was quantified by computing the variance of the relative hybridization intensity between allelic states as a percentage of the total variance of the relative hybridization intensity.

Markers with a quality parameter and a call rate both greater than 80% were selected to construct pilot linkage maps. Markers with a quality parameter between 75 and 80% were incorporated on a case-by-case basis (see section entitled "Pilot maps" below).

#### Other markers used for this study

The Barque-73/CPI71284-48 was typed with multiplex-ready SSR markers (Hayden, Nguyen and Chalmers, in preparation). The Dayton/Zhepi2 population was assayed with 38 SSR and STS markers as described previously (Raman et al., submitted) [53]. The TX9425/Franklin and Yerong/Franklin populations were typed with four SSR markers per chromosome according to Ramsay et al. [35]. The Clipper/Sahara and the Foster/CI4196 populations had previously been typed with SSR and/or RFLP markers [50,52].

### Data curation

#### DArT assay quality filtering

A total of 84 DArT assays from two populations had to be discarded because a subset of DNA samples stored in 96-well microtiter plates got cross-contaminated during shipment as a result of insufficient sealing of the plates. (These samples could be identified because of their bias toward "1" scores.) The remaining DArT assays were accepted if the relative hybridization intensities of non-polymorphic DArT markers were sufficiently correlated with the corresponding intensities in simultaneously performed assays (average correlation coefficient > 0.80). Application of this threshold led to the removal of 13 of the 966 remaining assays.

#### Quality filtering of other markers

A few markers with multiple entries in a single dataset were removed because the segregation signatures of the multiple entries did not resemble each other. The segregation data of other markers with multiple entries whose segregation signatures were almost identical (>95%) were collapsed into single-marker entries with consensus segregation signatures.

#### Marker nomenclature

DArT marker names are standardized and automatically generated by a DArT-specific Laboratory Information Management System (DArTdb; DArT P/L, Canberra, Australia). Additional File 4 contains a translation table between these names and the provisional names used in a previous paper [16]. Different laboratories used slightly different names for the same SSR or RFLP markers and assigned different letters to the multiple loci of multi-locus markers. Non-DArT marker names and locus codes, therefore, were curated to an extent required to create an unambiguous nomenclature (Additional File 4).

#### Merging the datasets

The presence vs. absence DArT scores (0/1) of the remaining 953 lines were converted into genotype codes (A/B/C/D) by comparison with the appropriate parental DArT assays (18 in total). Some markers for which both parental assays produced unreliable data were arbitrarily assigned to one of the two linkage phases. The segregation data for DArT markers were merged with those of other markers for each population for which non-DArT data was available.

#### Linkage group assignment

The segregation signatures of each of the ten individual datasets were imported into JoinMap 3.0 to distribute loci into linkage groups (Figure 1). The LOD threshold used to define linkage groups was necessarily dependent on the number of markers and lines in the datasets. Markers in the wrong linkage phase were identified and flipped into the opposite phase. The known chromosomal locations of a subset of the DArT markers [16] were used to assign linkage groups to chromosomes. Multi-dose markers were identified by comparing chromosomal assignment of markers across populations. Alternative loci were encoded by adding lower-case suffixes to marker names in the case of DArT markers.

#### Removal of redundant lines

Twenty redundant lines were identified with JoinMap 3.0 using a similarity threshold of 95%. They were removed from the datasets, thus reducing the total number of lines to 915.

#### Pilot maps

Pilot maps for individual populations were built by ordering loci with RECORD [26] to identify potential DNA sample-tracking errors between laboratories and to remove unreliable lines and loci (Figure 1). In the case of RIL lines, A/B (instead of C/D) genotype codes were used for map construction, assuming that residual heterozygosity levels were low. Inspection of the graphical genotypes identified a clear DNA sample-tracking inconsistency in one of the datasets: the scores of 14 of the 38 SSR loci were "frame-shifted" by one DH line with respect to the DArT data. The frame shift was rectified.

Lines with an excess of singletons were identified by inspecting graphical genotypes and removed from the corresponding dataset (4 of 915 lines). Loci that introduced a large number of singletons (typically two or more) were identified using a purpose-built perl script (Additional File 13). These loci were removed from the corresponding population dataset unless visual inspection of graphical genotypes showed that they were located in map regions with low marker densities. In this way, 86 of the 2,082 DArT loci (4.1%) with a quality parameter greater than 80% and 65 of the 956 (6.8%) non-DArT loci were completely removed from the combined (all-population) data set. Unidentified DNA sample-tracking inconsistencies between laboratories are likely to have caused the removal of a small number of RFLP or SSR loci during this process. A set of 41 closely linked markers with low call rates were removed from chromosome 2H of the Foster/CI4196 dataset. A limited number of DArT loci with a quality parameter of less than 80% were added to individual pilot maps if they introduced less than two singletons, which increased the total number of DArT loci by 89.

A comparison of the pilot maps across populations revealed three additional multi-locus DArT markers that mapped to different loci within the same chromosome. The loci were encoded as described in the section entitled "Linkage group assignment". The final curated dataset contained a total of 2,935 loci (2,085 DArT and 850 other loci) partially scored across 911 lines of ten populations. The quality of this dataset was later confirmed to be sufficient for building a high-quality consensus map (see *Results and Discussion*).

### Construction of component maps

Before constructing linkage maps of individual populations, quality-filtered datasets with sufficient numbers of markers and lines (seven in total) were separately collapsed into bins of co-segregating loci using a purpose-built perl script (Additional File 14). Only loci with a minimum of 50 genotype calls overlapping the consensus segregation signature of a bin were attempted to be added to a bin. The order of the bins, each represented by the locus with the highest call rate, was then optimized within each chromosome with RECORD. Kosambi map distances were calculated using a simplified version of the perl script described in the section entitled "Calculation of map distances" below (Additional File 15) [54]. This script applied the same distance calculation algorithm as JoinMap 3.0. The algorithm is insensitive to singletons, but can produce negative distance estimates for closely segregating bins because of the imprecision of recombination distance estimates for pairs of bins with missing calls. Negative estimates (totaling 0.4 ± 0.3% of the map lengths; mean ± SD across populations) were arbitrarily set to 0.001 cM to indicate closeness between bins and to retain the previously optimized bin order. The bins were then de-collapsed to reinsert co-segregating loci (Figure 1).

### Construction of a consensus map

#### DArT skeleton bin map

JoinMap 3.0 was used to build an initial draft of a consensus map based on the 1,546 'bPb' DArT loci collapsed into 959 bins. The program thresholds were adjusted to LOD = 2.0 and REC = 35 according to Karakousis et al. [9]. (An analysis of one of the chromosomes established that a LOD threshold of 3 did not significantly improve the results.) The consensus bin order was then improved manually in an iterative manner. The segregation data of all populations were arranged according to the consensus order in separate Excel spreadsheets and graphical genotypes were displayed in color using the conditional formatting option of the program. Starting at population one, misplaced bins with large numbers of singletons were moved within chromosomes to positions where they created less (frequently no) singletons. The modified consensus order was than imposed on the other nine datasets, and the resulting graphical genotypes were inspected to accept or reject the order. When a new position of a particular bin was rejected because it increased the number of singletons in other populations, alternative positions were tested across all populations until a better position than the original was found. Once each of the populations had been processed this way, the entire procedure was repeated until the overall number of singletons could not be reduced any further.

#### Insertion of other loci into the DArT framework

Other markers (separately collapsed into bins) and the 'bPb' DArT bins that JoinMap had failed to integrate were added to the DArT bin framework, using a purpose-built perl script (Additional File 13; Figure 1). The script used the DArT framework as a fixed scaffold and tested, for each new bin, all possible positions to select the one that produced the smallest number of singletons (frequently none) in the concatenated dataset. Singletons were identified by comparing each genotype call with the closest bracketing calls for the same line within a 15-bin window on either side. Once all new bins had been integrated this way, the order of multiple bins inserted at identical positions was resolved manually using the iterative procedure described in the previous section.

#### Calculation of map distances

Map distances between adjacent bins arranged in the order of the consensus map were calculated using a purpose-built perl script implementing the multi-point algorithm described by Lalouel (Additional File 15) [40] (also implemented in JoinMap 3.0). The segregation data of each population were separately converted into two-point recombination frequency estimates between all pairs of bins. In the Foster/CI4196, the Frederickson/Stander and the Patty/Tallon populations, the cumulative recombination frequencies of the RIL were converted into per-meiosis values according to Haldane and Waddington [55]. From the recombination frequency estimates, two matrices were generated for each population. The matrices contained pairwise LOD scores and Kosambi distance estimates for bin pairs separated by a recombination frequency of less than 35%. Both types of matrices were averaged and merged across populations to create two consensus matrices containing average distance estimates and average LOD scores (the numbers of lines assayed in each population were used as weights for averaging). The average distance estimates were then weighted with the squared average LOD scores and fed into a linear equation system that computed distances between adjacent bins by minimizing the overall deviation between empirical and computed average distance estimates [23,24,40].

#### Refinement of the map

As outlined under "Construction of component maps" above, the distance-calculation algorithm was expected to generate negative distance estimates for some closely segregating bins, because of the statistical uncertainty inherent in the data. Bins represented by loci only scored in a single population represented an additional source of negative distance estimates because their positions were inherently more ambiguous and to some degree still unresolved by our manual bin-ordering procedure.

The consensus bin order, therefore, was further refined with a purpose-built perl script that flipped the order of bin pairs with negative distance estimates until all negative estimates were eliminated (Additional File 15; Figure 1). Inspection of the resulting graphical genotypes showed that only few singletons were introduced by this procedure; most of them were due to a limited number of bins which the flipping procedure had shifted to sub-optimal positions. The positions of these bins were rectified manually, which inevitably re-introduced a limited number of negative distance estimates that were probably due to chromosome areas in which recombination frequencies differed among populations. Finally, the bins were de-collapsed to re-integrate co-segregating loci. Because this step introduced additional segregation data, which in some cases resolved the order of hitherto (almost) unresolved loci, a final round of manual improvement of the locus order was required (Figure 1). The remaining negative distance estimates, totaling 8.8% of the length of the consensus map, were set to 0.001 cM to indicate closeness and to retain the optimized bin order.

### Construction of a synthetic map from component maps

The locus positions from seven component maps with sufficient numbers of lines and loci (Table 3; Figure 2; Additional Files 1, 2) were merged to build a 'synthetic' map using PhenoMap software (GeneFlow Inc., Centreville, VA). Three analyses were performed, each using a different 'base' or reference map: Steptoe/Morex (largest number of loci), Yerong/Franklin (largest number of lines) and the map selected by PhenoMap as the one containing the largest number of common loci across populations (selected independently for each chromosome). The base map established the order for all common markers it contains; the remaining common markers were added in an iterative fashion by processing the remaining maps in descending order of the number of loci they have in common with the growing synthetic map. Once all common markers had been ordered, unique markers were added to the synthetic map. To place a unique marker, its relative distance to the nearest flanking common markers on the component map was calculated and scaled to the equivalent distance on the synthetic map.

## Authors' contributions

**PW **performed some DArT assays, reanalyzed all DArT array images, developed and tested various map construction strategies, wrote the perl scripts, built the component maps and the JoinMap/perl consensus map and drafted the manuscript, figures and additional files. **HL **produced a four-population pilot consensus map with JoinMap and prepared DNA samples and typed SSR markers for the TX9425/Franklin and the Yerong/Franklin population. **JC **performed most of the DArT assays. **MZ **developed DH populations from the Dayton/Zhepi2, the TX9425/Franklin and the Yerong/Franklin cross. **EP **built the synthetic maps with PhenoMap and edited the manuscript. **HR **compiled the information of marker-trait associations from the literature and mapped part of the SSR markers in the Dayton/Zhepi2 population. **PH **genotyped the multiplex-ready SSR markers for the Barque-73/CPI71284-48 population and co-developed an independent high-density map for this cross. **CM **performed most of the original RFLP mapping work for the Foster/CI4196 population. **LX **and **VC **performed some DArT assays. **JO **isolated DNA from the Igri/Atlas68 progeny and edited the manuscript. **MC **extracted DNA from the Patty/Tallon population and built an alternative map for this population. **DP **developed the Patty/Tallon population. **JW **isolated DNA from the Dayton/Zhepi2 population and produced the chromosome-4H SSR/STS data. **RR **mapped part of the SSR markers in the Dayton/Zhepi2 population.**KPS **and **GJM **co-developed the Frederickson/Stander DH population, built an alternative map for this population and edited the manuscript.**KJC **genotyped part of the SSR markers for the Barque-73/CPI71284-48 population and co-developed an independent high-density map for this cross. **AKleinhofs **built the original RFLP map of the Steptoe/Morex population and contributed to the mapping of RFLP markers in the Foster/CI4196 population. **EH **co-supervised and analyzed DArT assays and edited the manuscript. **AKilian **co-supervised the DArT assays, provided overall guidance during development, testing and implementation of DArT for barley, and co-designed and edited the manuscript. All authors read and approved the final manuscript.

## Supplementary Material

Additional file 1 **Component maps**. PDF file with graphical representations of individual maps. The maps were built separately for seven populations with sufficient numbers of markers and lines (Table 3). Click here for file

Additional file 2 **Locus positions in component maps**. Excel spreadsheet with the locus positions of the component maps. Click here for file

Additional file 3 **Relationship between DArT marker quality values and marker call rates**. PDF file with a plot of marker call rate vs. the quality value of DArT markers. Click here for file

Additional file 4 **Features of loci of the consensus map**. Excel spreadsheet containing a list of all consensus map loci and their features. Data include locus alias names (including those used by Wenzl et al. [16]]), the chromosome and position of each locus, the number of loci to which each marker was mapped, the number of populations in which each locus was mapped, and the across-populations average and standard deviation of the DArT locus quality parameter. Click here for file

Additional file 5 **Segregation data and graphical genotypes**. Excel file with ten spreadsheets, each containing the segregation data of a single population arranged according to the locus order of the consensus map. The genotype data are painted in colors to visualize the graphical genotypes underlying the maps. Click here for file

Additional file 6 **Consensus map**. PDF file with a detailed graphical representation of the consensus map including locus names. Click here for file

Additional file 7 **Statistics of consensus map by chromosome**. PDF file with a table containing chromosome-specific values for the number of loci, the number of bins, inter-bin distances and map lengths in cM. Click here for file

Additional file 8 **Multi-locus markers**. PDF file with a table containing the numbers of DArT and non-DArT markers that map to two or more loci. Click here for file

Additional file 9 **Influence of dataset composition on locus-ordering precision**. PDF file with two graphs showing the dependencies of (i) the correlation coefficients between two alternative sets of locus positions and (ii) the degree of SARF increase relative to individually optimized component maps, on the percentage of non-DArT loci in individual datasets. Click here for file

Additional file 10 **Number of 'bPb' DArT markers linked to trait-influencing loci on different chromosomes**. PDF file with a table containing the within-chromosome averages of the number of 'bPb' DArT markers in the vicinity of loci influencing agricultural traits. Click here for file

Additional file 11 **List of marker-trait associations**. Excel spreadsheet containing a list of loci influencing agricultural traits, including information on closely linked SSR, RFLP, STS and DArT markers. Click here for file

Additional file 12 **Distribution of loci affecting agricultural traits**. PDF file with a graphical representation of the consensus map in which only loci affecting agricultural traits and closely linked markers are highlighted. Click here for file

Additional file 13 **Perl script for integrating new loci**. Text file with perl code for integrating new loci into a fixed framework map. Execution of this file requires perl 5.8 to be installed first. Instructions for preparing the input files are displayed upon executing the program. An improved version of this script may be available from Peter Wenzl peter@DiversityArrays.com or Andrzej Kilian andrzej@DiversityArrays.com at DArT P/L [58]. Click here for file

Additional file 14 **Perl script for merging datasets and binning loci**. Text file with perl code for (i) merging segregation data of multiple populations and (ii) binning loci based on their segregation signatures concatenated across all populations. Execution of this file requires perl 5.8 to be installed first. Instructions for preparing the input files are displayed when executing the program. An improved version of this script may be available from Peter Wenzl peter@DiversityArrays.com or Andrzej Kilian andrzej@DiversityArrays.com at DArT P/L [58]. Click here for file

Additional file 15 **Perl script for computing map distances**. Text file with perl code for computing map distances for a given locus order from the segregation data of multiple populations based on the multipoint algorithm of Lalouel [40]. Execution of this file requires installation of perl 5.8 and the Math::Matrix perl module. Instructions for preparing the input files are displayed when executing the program. An improved version of this script may be available from Peter Wenzl peter@DiversityArrays.com or Andrzej Kilian andrzej@DiversityArrays.com at DArT P/L [58]. Click here for file
