# Supplementary material for: A high-density consensus map of barley linking DArT markers to SSR, RFLP and STS loci and agricultural traits
Source: BMC Genomics. 2006 Aug 12;7:206. doi: 10.1186/1471-2164-7-206 (PMC1564146; doi:10.1186/1471-2164-7-206)
Supplement: Additional file 1 — Component maps. PDF file with graphical representations of individual maps. The maps were built separately for seven populations with sufficient numbers of markers and lines (Table 3). [file 1471-2164-7-206-S1.pdf]

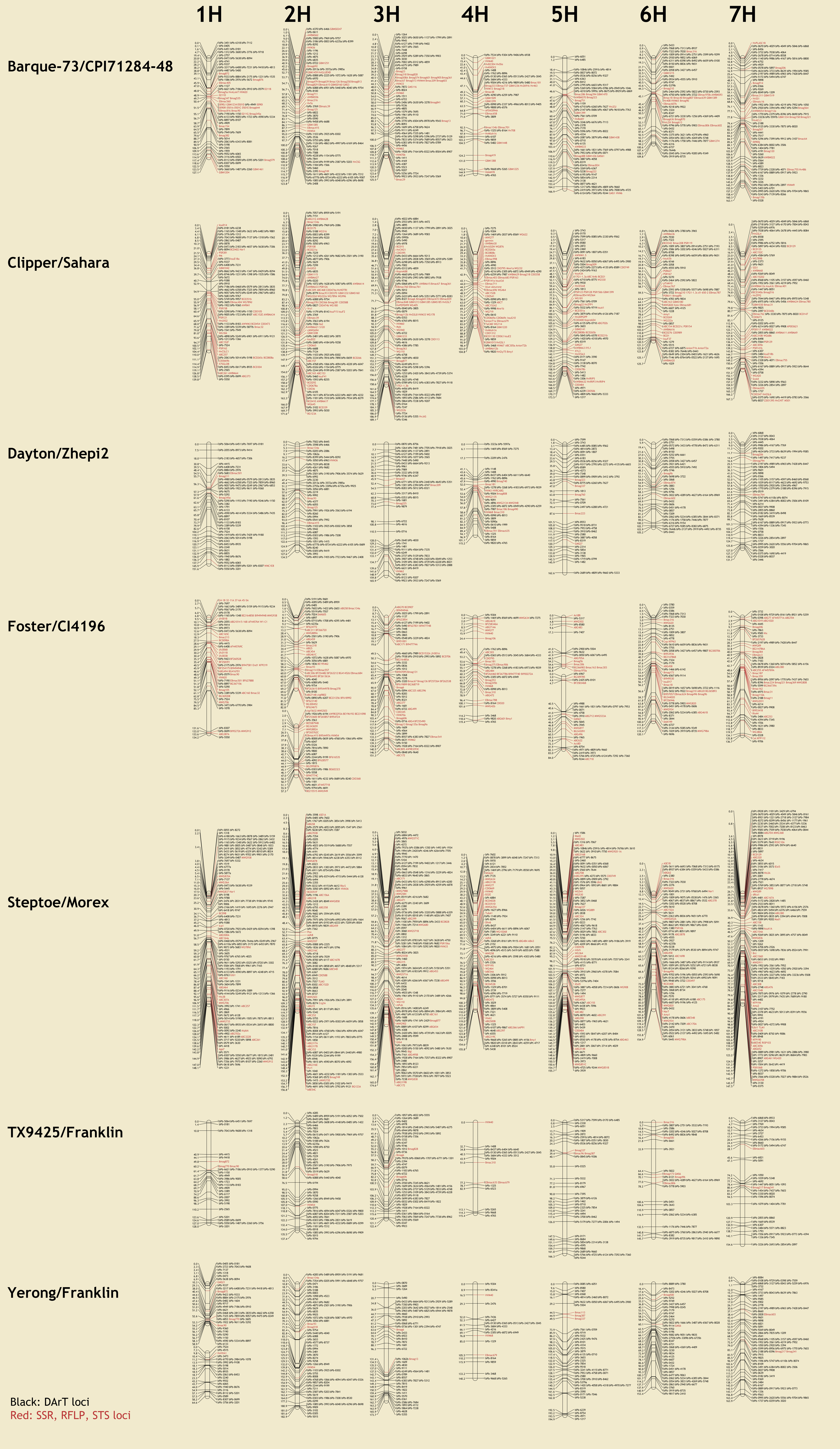

1H
2H
3H
4H
5H
6H
7H
Barque-73/CPI71284-48
Clipper/Sahara
Dayton/Zhepi2
Foster/CI4196
Steptoe/Morex
TX9425/Franklin
Yerong/Franklin
Black: DArT loci
Red: SSR, RFLP, STS loci
