# Supplementary material for: A high-density consensus map of barley linking DArT markers to SSR, RFLP and STS loci and agricultural traits
Source: BMC Genomics. 2006 Aug 12;7:206. doi: 10.1186/1471-2164-7-206 (PMC1564146; doi:10.1186/1471-2164-7-206)
Supplement: Additional file 3 — Relationship between DArT marker quality values and marker call rates. PDF file with a plot of marker call rate vs. the quality value of DArT markers. [file 1471-2164-7-206-S3.pdf]

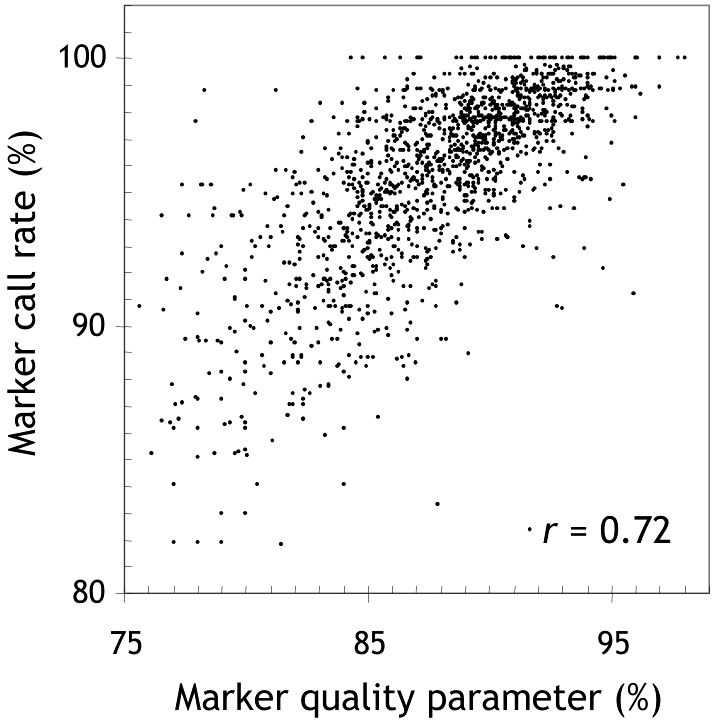

Additional file 3: ***Relationship between DArT marker quality values and marker call rates.*** The quality value of a marker is the between-allelic-states variance of the relative hybridization intensity as a percentage of the total variance of the relative hybridization intensity. Only the 'bPb' DArT markers from a *Pst*I/*Bst*NI representation were used to generate this graph.
