# Supplementary figures and images for: A high-density consensus map of barley linking DArT markers to SSR, RFLP and STS loci and agricultural traits
Source: BMC Genomics. 2006 Aug 12;7:206. doi: 10.1186/1471-2164-7-206 (PMC1564146; doi:10.1186/1471-2164-7-206)

# DArT/SSR/RFLP/STS consensus map

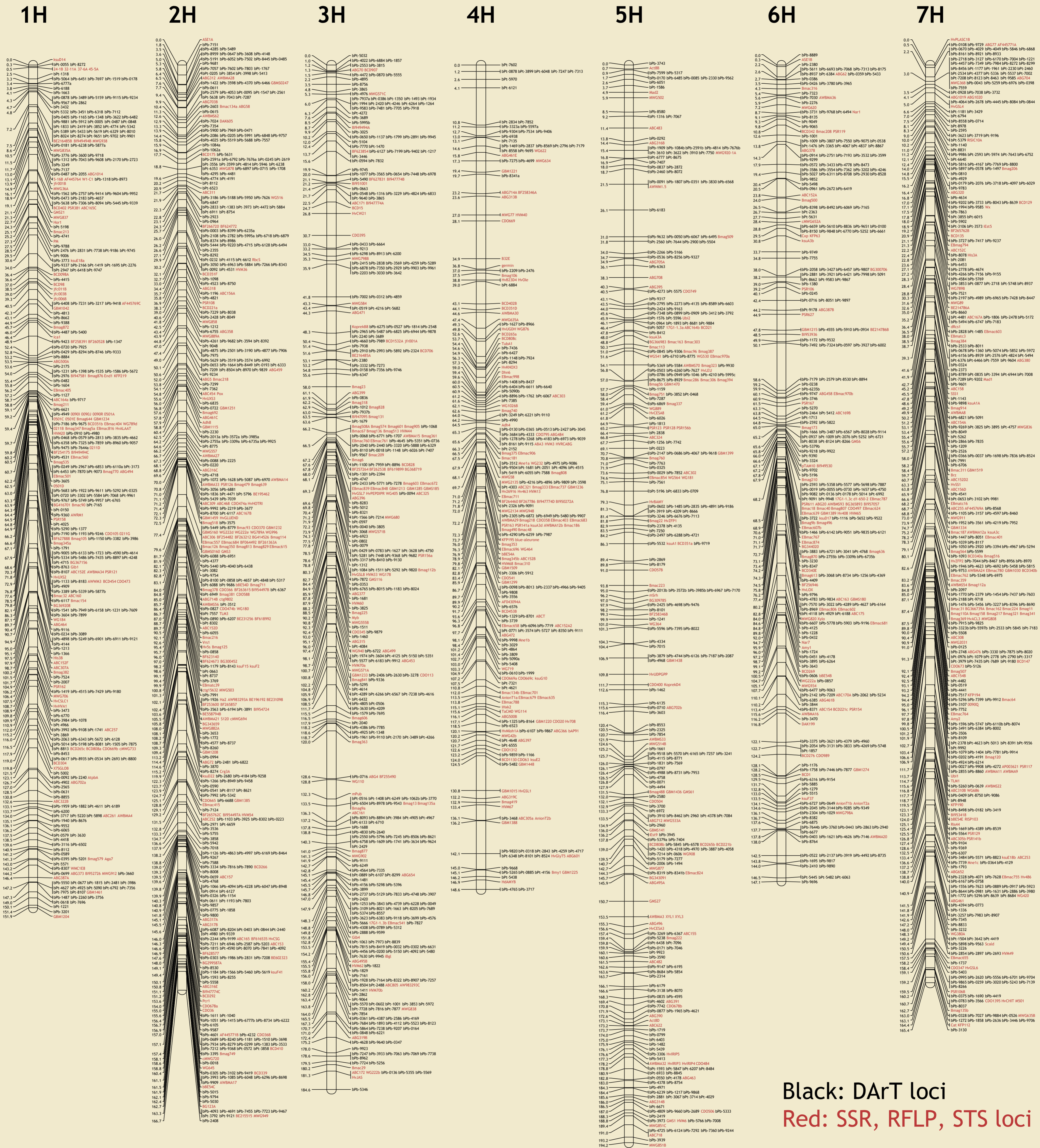

Black: DArT loci  
Red: SSR, RFLP, STS loci

Supplement: Additional file 6 — Consensus map. PDF file with a detailed graphical representation of the consensus map including locus names. [file 1471-2164-7-206-S6.pdf]

# Associations between ‘bPb’ DArT markers and agricultural traits

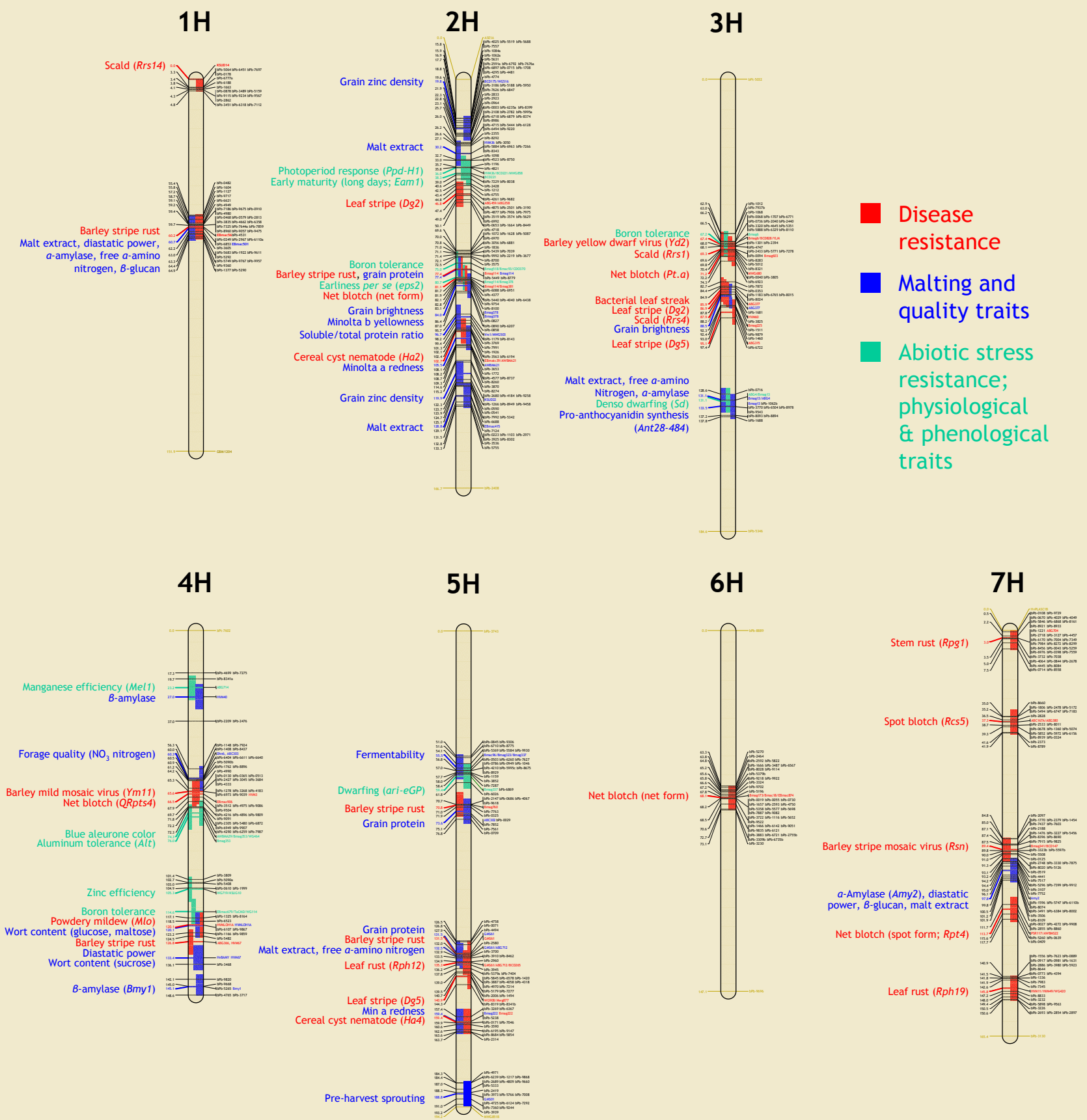

Supplement: Additional file 12 — Distribution of loci affecting agricultural traits. PDF file with a graphical representation of the consensus map in which only loci affecting agricultural traits and closely linked markers are highlighted. [file 1471-2164-7-206-S12.pdf]
