# Supplementary material for: A high-density consensus map of barley linking DArT markers to SSR, RFLP and STS loci and agricultural traits
Source: BMC Genomics. 2006 Aug 12;7:206. doi: 10.1186/1471-2164-7-206 (PMC1564146; doi:10.1186/1471-2164-7-206)
Supplement: Additional file 7 — Statistics of consensus map by chromosome. PDF file with a table containing chromosome-specific values for the number of loci, the number of bins, inter-bin distances and map lengths in cM. [file 1471-2164-7-206-S7.pdf]

Additional File 7: Statistics of consensus map by chromosome.

Map features were calculated for the whole dataset and various subsets obtained by removing selected groups of loci while maintaining the locus order of the consensus map.

	1H	2H	3H	4H	5H	6H	7H	Genome
**Number of loci**								
**All**	423	522	470	278	379	362	501	2,935
**DArT**	301	362	361	148	270	270	373	2,085
**'bPb' DArT**	212	276	255	91	213	219	280	1,546
**Others**	122	160	109	130	109	92	128	850
**Number of bins**[a]								
**All**	225	298	246	162	241	191	266	1,629
**DArT**	154	206	190	82	169	140	189	1,130
**'bPb' DArT**	130	178	158	58	143	127	165	959
**Others**	81	108	73	92	82	66	92	594
**Average/median inter-bin distance (cM)**[a]								
**All**	0.7/0.3	0.6/0.3	0.8/0.3	0.9/0.4	0.8/0.5	0.8/0.3	0.6/0.3	0.7/0.3
**DArT**	1.0/0.4	0.8/0.3	1.0/0.3	1.8/1.0	1.2/0.6	1.1/0.4	0.9/0.3	1.0/0.4
**'bPb' DArT**	1.2/0.4	0.9/0.4	1.7/0.3	2.4/1.3	1.4/0.7	1.2/0.4	1.0/0.3	1.2/0.4
**Others**	1.9/1.3	1.5/1.0	2.5/2.0	1.5/1.0	2.4/1.9	2.1/1.4	1.8/1.1	1.9/1.3
**Map length (cM)**								
**All**	151.9	166.7	184.6	148.6	194.2	147.1	165.4	1,159
**DArT**	151.1	164.9	184.6	148.6	193.2	147.1	165.0	1,155
**'bPb' DArT**	148.1	161.6	183.6	137.4	193.2	147.1	165.0	1,136
**Others**	151.9	163.3	180.0	132.3	193.5	137.5	164.4	1,123

[a] Segregation signatures were concatenated across populations and co-segregating loci were collapsed into bins (unique loci) based on a minimum of 50 overlapping genotype calls (see *Methods* section entitled "Construction of component maps").
