# Supplementary material for: A high-density consensus map of barley linking DArT markers to SSR, RFLP and STS loci and agricultural traits
Source: BMC Genomics. 2006 Aug 12;7:206. doi: 10.1186/1471-2164-7-206 (PMC1564146; doi:10.1186/1471-2164-7-206)
Supplement: Additional file 8 — Multi-locus markers. PDF file with a table containing the numbers of DArT and non-DArT markers that map to two or more loci. [file 1471-2164-7-206-S8.pdf]

Additional File 8: Multi-locus markers.

No. loci mapped	DArT markers	Other markers
2	21	48
3	1	10
4	0	6
5	0	1
> 5	0	1
