# Supplementary material for: A high-density consensus map of barley linking DArT markers to SSR, RFLP and STS loci and agricultural traits
Source: BMC Genomics. 2006 Aug 12;7:206. doi: 10.1186/1471-2164-7-206 (PMC1564146; doi:10.1186/1471-2164-7-206)
Supplement: Additional file 9 — Influence of dataset composition on locus-ordering precision. PDF file with two graphs showing the dependencies of (i) the correlation coefficients between two alternative sets of locus positions and (ii) the degree of SARF increase relative to individually optimized component maps, on the percentage of non-DArT loci in individual datasets. [file 1471-2164-7-206-S9.pdf]

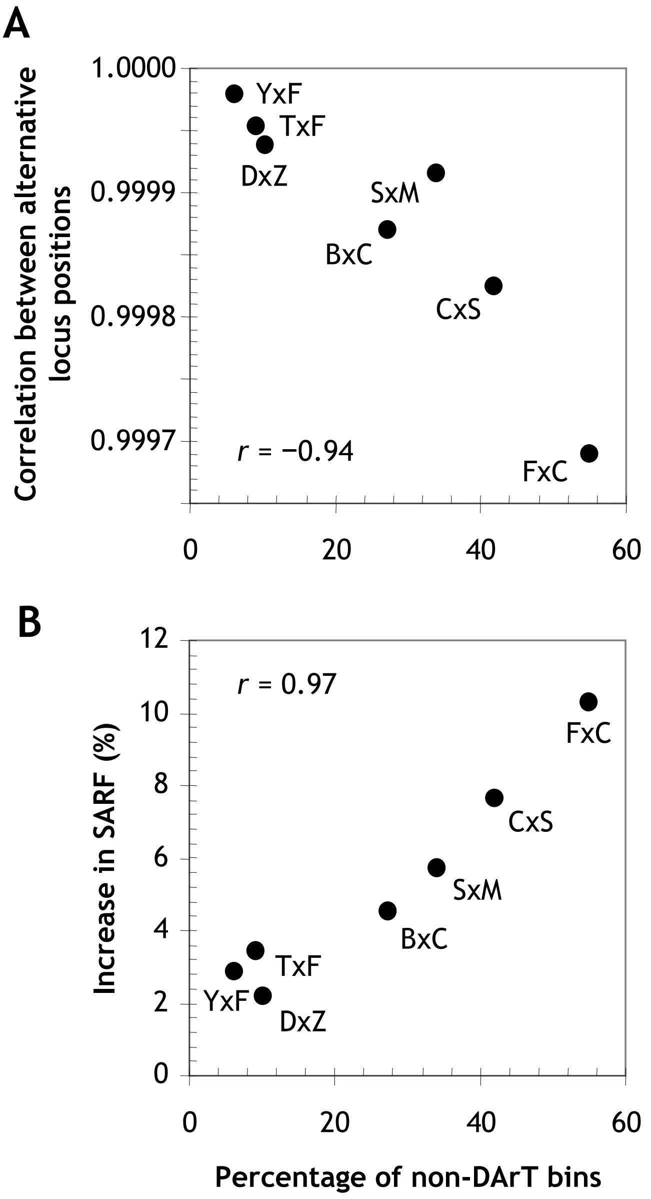

Additional File 9 - Influence of the dataset composition on locus-ordering precision. (A) Similarity between the arrangement of loci in the consensus map and their arrangement in individual component maps. The across-chromosomes average of the correlation coefficient between two alternative sets of locus positions, reflecting either consensus or component-map order, is shown as a function of the percentage of non-DArT loci in the individual datasets. **(B)** Map expansion caused by imposing the consensus locus order on individual datasets. The increase in SARF compared to individually optimized component maps is shown as a function of the percentage of non-DArT loci in individual datasets. Abbreviations of populations: B/C, Barque-73/CPI71284-48; C/S, Clipper/Sahara; D/Z, Dayton/Zhepi2; F/C, Foster/CI4196; S/M, Steptoe/Morex; T/F, TX9425/Franklin; Y/F, Yerong/Franklin.
