# Supplementary material for: A high-density consensus map of barley linking DArT markers to SSR, RFLP and STS loci and agricultural traits
Source: BMC Genomics. 2006 Aug 12;7:206. doi: 10.1186/1471-2164-7-206 (PMC1564146; doi:10.1186/1471-2164-7-206)
Supplement: Additional file 10 — Number of 'bPb' DArT markers linked to trait-influencing loci on different chromosomes. PDF file with a table containing the within-chromosome averages of the number of 'bPb' DArT markers in the vicinity of loci influencing agricultural traits. [file 1471-2164-7-206-S10.pdf]

Additional File 10: Average number of ‘bPb’ DArT markers within 5 cM on either side of non-DArT markers previously identified to be associated with phenotypic traits.

	Number of linked ‘bPb’ DArT markers		
**Chromosome**	**Average ± SD**	**Range**	**Number of trait-influencing loci on chromosome**
1H	30 ± 11	17 – 37	3
2H	12 ± 6	5 – 25	17
3H	14 ± 9	4 – 27	12
4H	8 ± 8	0 – 26	15
5H	13 ± 4	7 – 21	12
6H	41	41	1
7H	22 ± 10	6 – 35	6
